# The role of faecal microbiota transplantation: looking beyond *Clostridioides difficile* infection

**DOI:** 10.1177/2049936120981526

**Published:** 2021-01-25

**Authors:** Simon D. Goldenberg, Blair Merrick

**Affiliations:** Centre for Clinical Infection & Diagnostics Research, King’s College London and Guy’s & St. Thomas’ NHS Foundation Trust, 5th floor, North Wing, St Thomas’ hospital, Westminster Bridge Road, London, SE1 7EH, UK; Centre for Clinical Infection & Diagnostics Research, King’s College London and Guy’s & St. Thomas’ NHS Foundation Trust, London, UK

**Keywords:** faecal microbiota transplantation (FMT), clostridioides difficile infection (CDI), dysbiosis, inflammatory bowel disease (IBD), irritable bowel syndrome (IBS), liver disease

## Abstract

Faecal microbiota transplantation (FMT) is the transfer of screened and minimally processed faecal material from a ‘healthy’ donor to ‘diseased’ recipient. It has an established role, and is recommended as a therapeutic strategy, in the management of recurrent *Clostridioides difficile* infection (CDI). Recognition that gut dysbiosis is associated with, and may contribute to, numerous disease states has led to interest in exploiting FMT to ‘correct’ this microbial imbalance. Conditions for which it is proposed to be beneficial include inflammatory bowel disease, irritable bowel syndrome, liver disease and hepatic encephalopathy, neuropsychiatric conditions such as depression and anxiety, systemic inflammatory states like sepsis, and even coronavirus disease 2019. To understand what role, if any, FMT may play in the management of these conditions, it is important to consider the potential risks and benefits of the therapy. Regardless, there are several barriers to its more widespread adoption, which include incompletely understood mechanism of action (especially outside of CDI), inability to standardise treatment, disagreement on its active ingredients and how it should be regulated, and lack of long-term outcome and safety data. Whilst the transfer of faecal material from one individual to another to treat ailments or improve health has a history dating back thousands of years, there are fewer than 10 randomised controlled trials supporting its use. Moving forward, it will be imperative to gather as much data from FMT donors and recipients over as long a timeframe as possible, and for trials to be conducted with rigorous methodology, including appropriate control groups, in order to best understand the utility of FMT for indications beyond CDI. This review discusses the history of FMT, its appreciable mechanisms of action with reference to CDI, indications for FMT with an emerging evidence base above and beyond CDI, and future perspectives on the field.

## A brief history of faecal microbiota transplantation

The first documented use of the ingestion of faecal material to treat illness comes from the Sanskrit text *Charak Samhita. Panchgavya*, a mixture of five cow products including dung, used in ayurvedic medicine for over 2000 years, claims to treat a variety of communicable and non-communicable diseases.^1^ It is unsurprising that consumption of products from a sacred animal was thought to improve health, nevertheless, a substantiated evidence base is lacking.

Chinese literature from the 4th century describes faecal material being used to treat food poisoning and diarrhoeal illnesses, and again in the 16th century, ‘golden syrup’, a mixture of faeces and water, was used with medicinal intent to cure abdominal illnesses.^2^ In Korea, ancient texts describe *Ttsongul*, a fermented rice wine made using faeces, believed to be effective in treating a wide range of problems from cuts through to epilepsy.^3^ Throughout other parts of Asia and the Middle East, Bedouins have treated dysentery with fresh camel dung for centuries.^4^ However, evidence as to the efficacy of any of these is at best anecdotal.

Faecal microbiota transplantation (FMT) first entered modern medicine in 1958 when Eiseman and colleagues successfully treated four patients with pseudomembranous colitis using faecal enema.^5^ Numerous case series and open-label trials followed with FMT used to treat inflammatory bowel disease (IBD) as well as and CDI (now known to be the predominant cause of pseudomembranous colitis), but it was not until 2013, that the first randomised control trial (RCT) evidence of FMT efficacy in the treatment of recurrent (r) CDI was published.^6^ The trial was stopped after an interim analysis demonstrated clear benefit of FMT compared with vancomycin. Subsequent RCTs and meta-analyses have confirmed that FMT (whether fresh, frozen or lyophilised, and given orally, *via* endoscopy or enema), is superior to antibiotics for treating relapsed or refractory CDI.^7–13^ Although most guidelines do not recommend FMT for treatment of any conditions other than rCDI,^14^ interest in using FMT for other indications is growing. Here, we review proposed mechanisms of action of FMT with a focus on CDI, the current evidence base for other indications, and consider future perspectives on the direction the field of FMT may take.

## The constituents of FMT and proposed mechanisms of action

Microbiota is the descriptive term for all the organisms found in a particular niche, such as the human gut, including bacteria, viruses, archaea, and protozoa. The microbiome is the sum of the genetic material of these organisms. Modern sequencing techniques have enabled us to ‘see’ these organisms, as well as their relative abundance.^15^ The microbiota is a dynamic entity and varies not only from person to person (alpha diversity), but also over time in individuals (beta diversity). Metabolomic profiling, the ‘fingerprint’ left behind by the activities of the microbiota and its interaction with the host, adds further detail to our knowledge of this highly complex environment.^16,17^ Studies undertaken in health and in disease states have identified differences in all of these parameters.^18^ Deviations from the ‘norm’ have been termed dysbiosis (although this term has been criticised by some^19^) and can be seen locally, that is, within the gut, as well as systemically, for example, changes in lymphocyte subsets. The ability of FMT to ‘correct’ dysbiosis, or this ‘imbalance’, is of great interest.

**Figure 1.**
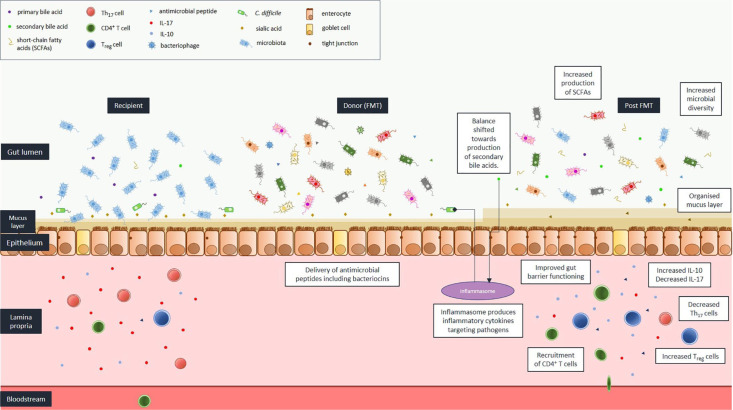
Mechanisms of action of faecal microbiota transplantation (FMT) with reference to *Clostridioides difficile* infection (CDI). *C. difficile, Clostridioides difficile*; IL, interleukin; SCFA, short-chain fatty acid; TH_1_, T-helper cell 1; Treg cell, regulatory T cell.

In CDI, there is, almost invariably, a recent alteration in gut microbiota following antimicrobial administration (usually several days to weeks before developing infection). For other disease states, for example, IBD, dysbiosis is often a more established process (months to years). This is an important difference, as FMT efficacy rates, and optimal dosing regimens may vary depending on the duration of dysbiosis. The proposed mechanisms of action of FMT, with specific reference to CDI, are depicted in Figure 1 and discussed below. The relative contribution of each is currently unknown. None, some, or all these mechanisms may be applicable to the activity of FMT for non-CDI conditions.

### Colonisation resistance

Colonisation resistance is the protection afforded by healthy microbiota against invading pathogens. Antimicrobials are taken, for the most part, to kill, or at least prevent growth, of pathogenic microbes. Taken systemically, collateral disruption to the body’s microbiota occurs. Destabilising microbial communities disrupts microbiota-mucosal immunity interactions and promotes inflammation, allowing sites such as the gut to become colonised with pathogens including *C. difficile*. Ingestion of *C. difficile* spores leads to asymptomatic carriage, or in the worst-case scenario, to disease (CDI). Replenishing and diversifying the microbial community using FMT promotes colonisation resistance.^20^ A trend towards increased bacterial community diversity in recipients successfully treated with FMT suggests causality.^21^

### Gut barrier

A healthy gut microbiota provides constant stimulation to the epithelial barrier leading to the production of an organised mucus layer, the maintenance of tight junctions between epithelial cells, and the production of antimicrobial peptides by the host. All these actions serve to compartmentalise the microbiota within the intestinal lumen. Disrupted microbiota-host crosstalk in dysbiosis means the integrity of the gut barrier is reduced, and in the most extreme cases, for example pseudomembranous colitis, there is total breakdown with translocation of residual microbiota. FMT can prevent, stabilise, and even reverse these effects.^22^

### Bile acids

Bile acids are capable of directly influencing the microbiota and have been demonstrated to have antimicrobial activity,^23,24^ including affecting the growth of *C. difficile*. Primary bile acids (those initially released from the duodenum) stimulate germination of spores, whereas secondary bile acids (formed *via* 7α-dehydroxylation of primary bile acids by *Lacnospiraceae* and *Ruminococcaceae* families) inhibit this process.^25^ In faeces, secondary bile acids have been noted to be present in lower levels in individuals with CDI (especially those with recurrent disease) compared with those without, and FMT has been demonstrated to shift the balance from primary towards secondary bile acid synthesis in recipients.^26^ Bile acids interact with the host inflammasome, a multi-protein intracellular complex that detects pathogens, and induces the production of pro-inflammatory cytokines interleukin 1β (IL-1β) and IL-18. Inflammasome signalling is vital in recovery from CDI.^27^ Bile acids may also mitigate the effect of *C. difficile* exotoxins, thereby reducing epithelial damage.

### Nutrients/carbohydrates (including short-chain fatty acids)/proteins (e.g. metal chelation)

The inner and outer mucous layers of the gut are made of complex carbohydrates, which are a ready source of energy for bacteria that possess the necessary digestive enzymes to break them down into compounds such as glucose and sialic acid. Loss of the bacteria with antimicrobial treatment, which efficiently scavenge these products, leaves them available for pathogens such as *C. difficile*, allowing germination and proliferation.^28^

Short-chain fatty acids (SCFAs) are a group of carbohydrates produced by anaerobic fermentation in the gut from dietary fibre and resistant starch, with acetate, propionate and butyrate being the most abundant.^29^ Their presence has been linked with improvement in gut barrier functioning and reduced inflammation by supporting peripheral regulatory T (T_reg_)-cell development.^30^ Alterations in levels of SCFAs, or bacteria which produce SCFAs, have been detected between individuals with CDI and healthy controls. Medium-chain fatty acids are predominantly derived from triglycerides and phospholipids in plant oils and milk products. Some of these have been shown to have antimicrobial activity, for example, lauric acid, inducing oxidative damage and cell lysis in *C. difficile*. Contribution to the efficacy of FMT still remains unclear.^31^

Certain proteins, for example, calprotectin, are capable of binding metal ions, for example, zinc, reducing their availability. Zinc (as well as calcium) are co-factors for bacterial enzymes, and transfer of faecal material which reduces free metal ion availability in the recipient may prevent the development of CDI by interfering with enzyme activity.^32^

### Antimicrobial peptides

As well as competing with each other for nutrients, bacteria are capable of producing bacteriocins, antimicrobial proteins which target other, especially phylogenetically similar, bacterial species.^33^ Bacteriocins produced by Gram-positive bacteria are split into one of four classes; I [lantibiotics: small (<5 kDa) post-transcriptionally modified peptides with unusual amino acids], II [non-lantibiotics: small (<10 kDa) limited post-transcriptional modification and no unusual amino acids], III [large (>30 kDa), heat-labile lytic or non-lytic peptides], and IV (contain lipid or carbohydrate parts). Bacteriocins produced by Gram-negative bacteria include colicins (>10 kDa, produced by *Escherichia coli*), colicin-like (>10 kDa, produced by bacteria other than *E. coli*), microcins (molecular weight <10 kDa), and phage tail-like (very similar to phage tail structure). Further detail on their biology and spectrum of antimicrobial activity has recently been summarised.^34^

Several compounds that target *C. difficile* have been identified, including fidaxomicin, a drug given clinically to treat *C. difficile*. This is produced by the actinomycete, *Dactylosporangium aurantiacum* subspecies *hamdenesis*.^35^ Intestinal and immune cells have also been shown to produce compounds with antimicrobial activity such as defensins, cathelicidins and lysozyme.^36^

### Bacteriophages

Bacteriophages are enveloped ribonucleic acid (RNA) or deoxyribonucleic acid (DNA) viruses that bind specific receptors (and thereby determine host specificity) on bacteria and archaea.^37^ They are capable of inducing cell lysis, and modifying virulence factors or gene expression, including in *C. difficile*.^38^ They have been utilised to treat antimicrobial-resistant organisms (AROs) where other lines of therapy have failed.^39^

### Immunomodulation

The potential influence of FMT on the immune system was considered briefly above with respect to bile acids and SCFAs. Bacteria, too, have immunomodulatory actions, for example, *Bacteroides fragilis*, through its presentation of polysaccharide A (PSA), contributes to the maintenance of CD4^+^ T-lymphocyte development, balancing T-helper cell 1 (Th_1_) and Th_2_ responses, and lymphoid organogenesis. It can also inhibit the production of pro-inflammatory IL-17 (by inhibiting the Th_17_ response) and promote the production of anti-inflammatory IL-10 (by promoting the T_reg_ cell response).^40^ Further description of the relationship between the immune system and microbiota is beyond the scope of this paper, but has recently been summarised elsewhere.^41^ Needless to say, the importance of a commensal microbiota in the development and maintenance of a functioning immune system is without question, as demonstrated by the defects in both gastrointestinal (GI) and systemic immunity in germ-free animal models.^42,43^

## Regulation and safety of FMT

The regulatory landscape of FMT in the European Union and other jurisdictions has been reviewed elsewhere.^44^ There are widely varying approaches to its regulation and governance, with merits and disadvantages to each. As with any medical treatment, the benefits the patient stands to gain must be weighed against the risks of receiving FMT.

Of note, there has been a number of recent safety alerts relating to improperly screened donations which have resulted in transmission of infection.^45^ In light of the coronavirus disease 2019 (COVID-19) pandemic, donor material must now be screened for severe acute respiratory syndrome coronavirus 2 (SARS-CoV-2), as the virus is detectable and culturable from the stool of infected individuals, so theoretically transmissible *via* FMT.^46^ FMT regulation must be carefully considered going forward, to ensure both the delivery of a safe product and the availability of the treatment to clinicians in a clinical and research capacity.

## Indications with emerging evidence base

### Severe (primary) CDI

Traditional management of severe CDI refractory to antibiotics has been colectomy. This is associated with high fatality rates, especially in the frail and elderly.^47^ The success of FMT for recurrent and relapsed CDI led to the trialling of FMT in severe and fulminant infection with excellent results. Overall clinical cure at 1 month was achieved in 91% patients in one trial, and 88% at 8 weeks in another, with a suggestion that multiple infusions may be superior to just one (cure rate 100% *versus* 75%). There were no serious adverse events related to FMT or its delivery.^48,49^ Expert opinion recommends consideration of FMT early in these cases,^50^ but at present there remains insufficient RCT evidence to support its use. However, it would be impractical and potentially unethical to recruit these critically unwell patients into trials to get a definitive answer, given the overwhelming evidence of benefit in rCDI.

### Inflammatory bowel disease

There is emerging evidence that FMT can induce remission in ulcerative colitis (UC), one of the two main subtypes of IBD. Although encouraging, with success rates ranging from 25% to 45%,^51,52^ results fall well short of that seen in CDI. Individuals with IBD have reduced numbers of SCFA-producing obligate anaerobes and an increase in pro-inflammatory facultative anaerobes including *E. coli*.^53^ Interestingly, FMT from a donor with high colony counts of *Ruminococcaceae*, an obligate anaerobe and one of the main producers of butyrate in the gut, was more successful in inducing remission (39%) in UC compared with the other five donors (10%) in the study.^54^ These are observational data only and do not prove causality. It also does not explain why the majority (61%) failed to respond. The first RCT of FMT in Crohn’s disease (CD), the other main subtype of IBD, did not identify donor-specific response rates; however, sample size was small (21 participants). It did show a trend towards FMT promoting remission and a reduction in gut inflammation following steroid therapy, particularly if the recipient’s microbiome reflected that of their donor, compared with control, but results did not reach statistical significance.^55^ Larger studies are keenly anticipated. Defects in immune regulation including a reduction in T_reg_ lymphocytes and enhanced Th_17_ and innate cell response associated with the production of pro-inflammatory cytokines have also been implicated in the pathogenesis of IBD. As described above, these can be moderated by certain bacteria or their components, which can be transmitted in FMT.

### Primary sclerosing cholangitis

Primary sclerosing cholangitis (PSC) is a chronic, progressive cholestatic hepatobiliary disorder characterised by fibrotic destruction of bile ducts. It leads to biliary cirrhosis, portal hypertension and ultimately, liver failure. Concurrent IBD is common (80% patients), although the dysbiosis seen is distinct even from that seen in patients with IBD alone.^56^ It is proposed that bacterial translocation across an inflamed gut drives an inflammatory process in the biliary tree, leading to disease. This theory is supported in animal models.^57^ Attempts to modulate the gut microbiota of patients with PSC has been trialled with antimicrobials with mixed results, and it is not currently recommended as a therapeutic strategy.^58,59^ An open-label pilot study published last year demonstrated biochemical improvement (alkaline phosphatase levels fell by 50%) in recipients (3/10) of FMT in which there was donor engraftment out to 6 months post-transplant.^60^ The slowly progressive nature of disease means that long-term follow up will be necessary in future trials to establish any effect of FMT on patient outcomes.

### Irritable bowel syndrome

Irritable bowel syndrome (IBS) is a heterogeneous chronic GI condition affecting around 1 in 10 people worldwide. Dysbiosis (for example high *Firmicute: Bacteroidetes* ratio) has been identified in many individuals, making correction with FMT an attractive proposition. Pooled results from RCTs do not show a conclusive benefit to date. However, if the delivery method of FMT is considered, there is a suggestion fresh or frozen donor stool may be beneficial, whereas capsulised FMT may cause harm.^61–63^ Holvoet *et al.* suggested microbiota modulation with FMT should be targeted at the subgroups with severe bloating and flatulence, in whom there is the most significant disturbance in gut microbial composition. They believe donor selection is important, short-lived effects can be augmented by repeat FMT delivery, but that certain individuals may be refractory to FMT. The study raises important questions: why should there be differences in response rates by sex (females were more likely to respond)? And, why individuals with a greater baseline diversity in microbes pre-FMT were more likely to have a positive response to FMT.^64^ This would seem counterintuitive if FMT worked by increasing microbial diversity; potentially other transplanted components were the reason for efficacy. Another recently published trial agrees that donor selection is important but suggests that efficacy can be achieved across IBS subtypes, that response is dose dependent, and is related to improving dysbiosis in the recipient.^65^ Patients in this trial had a lower IBS-SSS (symptom severity score) than the Dutch study and follow-up was limited to 3 months, so how durable the effect on symptoms is unclear. A further study suggests that despite longlasting microbiota changes, clinical improvement is small and transient with no significant effect on quality-of-life outcomes.^66^

Lastly, individuals with both IBS and chronic fatigue syndrome (CFS), also known as myalgic encephalomyelitis (ME), were assigned to receive either oral approaches (e.g. pre/probiotics, dietary advice, nutritional remedies) or anaerobically produced FMT delivered into the sigmoid colon *via* rectal catheter from 10 different donors in a non-randomised fashion. FMT recipients improved to a greater extent than those who received oral approaches alone according to the investigators’ assessment, suggesting a potential role for FMT in CFS.^67^ However, the retrospective nature of the study, and the lack of randomisation and an objective standard for assessing improvement, limit the generalisability of results.

### Constipation

Slow-transit constipation (STC) is a common functional GI disorder and represents a subset of up to 30% individuals who suffer with constipation.^68^ Promising pilot study results with clinical improvement in 50–60% of participants up to 12 weeks^69,70^ were followed by the only RCT^71^ reported to date of FMT to treat STC, which again, showed favourable outcomes. Compared with control, the intervention group showed reduced transit time, and improved stool consistency. Overall, clinical improvement was seen in 53% of the intervention group (*versus* 20% in controls) and clinical cure in 37% (*versus* 13% in controls). However, the FMT regimen was intense (100 ml given daily for 6 days *via* naso-intestinal route) with a high rate of reported adverse events, and the study was not blinded, limiting attribution of the effect to FMT alone, as well as the broader application of results. FMT may also have a role in other types of constipation; an open-label study in patients with chronic intestinal pseudo-obstruction suggested that FMT could alleviate symptoms of pain and bloating.^72^

### Eradication of antimicrobial-resistant organisms

It was noted that patients treated with FMT for rCDI who had concurrent gastrointestinal colonisation with various AROs cleared these organisms following the procedure.^73,74^ This sparked interest that FMT could be used to eradicate AROs from the gut. ARO colonisation rates in the community are low in Europe and America, but much higher in Africa and Asia^75^ and in healthcare settings.^76^ Travel (or hospital admission) to these areas is a risk factor for ARO colonisation, even in ‘healthy’ individuals. Once exposure is removed, a fully functioning gut microbiota appears to prevent persistent engraftment. However, antimicrobial use, or medical comorbidities, both of which contribute to continued dysbiosis, may allow resistant organisms to persist. Correcting imbalance with FMT seems a logical therapeutic strategy. Research to date, has been hampered by numerous factors. First, lack of RCTs: uncontrolled studies take no account for the spontaneous loss of ARO carriage and there is also implicit risk of publication bias against negative results; and second, the concurrent use of antimicrobials prior to FMT makes it is difficult to attribute causality to FMT alone.^77–79^

### Immune-checkpoint-inhibitor-mediated colitis

Immune-checkpoint inhibitors are revolutionising the field of immuno-oncology, improving outcomes in a wide range of malignancies. A side effect of therapy is a colitis resembling IBD, both endoscopically and histologically, which usually responds to immunomodulatory treatment. Reports of refractory cases being successfully treated with FMT are emerging.^80,81^ One of these studies suggested possible mechanisms of action including FMT re-establishing the population of T_reg_ cells in the gut mucosa, as well as gut microbiota changes away from pathogenic species such as *Escherichia* towards species such as *Verrucomicrobiae* with anti-inflammatory activity through the production of SCFAs.

### Cirrhosis

The majority of research to date has focused on the prevention of hepatic encephalopathy, the most frequent manifestation of decompensated liver disease. RCTs have highlighted the safety and potential efficacy of FMT in improving both cognition and reducing hospital admissions in treatment *versus* control groups.^82,83^ Pre-treatment with antimicrobials does not appear necessary for FMT engraftment, and may actually cause harm (model for end-stage liver disease score transiently worsened post-antibiotics, but reverted to baseline post-FMT in one study).^82^ Studies reported to date have been limited by size, and included patients at the more severe end of the disease spectrum, so wider applicability remains unknown. FMT responders initially had an increase in secondary bile acids, possibly due to colonisation with *Ruminococceae* and *Lachnospiraceae*, and a rise in bacterial species such as *Verrucomicrobiae*, associated with the production of SCFAs. There was also evidence of reduced peripheral markers of inflammation (e.g. IL-6). However, by 12 months, many of these changes did not persist (although differences remained detectable between intervention and control groups); regardless, outcomes remained improved in terms of cognitive functioning and requirement for hospitalisation in patients who received FMT.^84^

### Alcoholic hepatitis

As the name suggests, alcoholic hepatitis (AH) is liver inflammation secondary to excessive alcohol consumption. It is usually seen in chronic heavy drinkers and is frequently a precursor to the development of cirrhosis. In the extreme, acute cases can be life threatening, with complications including jaundice, ascites and hepatic encephalopathy. Current treatment options such as corticosteroids and liver transplantation are limited by inefficacy and patient ineligibility. Data are starting to emerge to suggest FMT may have a role to play in the management of AH, with a couple of small studies showing reduction in mortality (80% survival in intervention groups *versus* 30–40% in control groups) and disease-associated complications, including ascites and hepatic encephalopathy, up to 1 year of follow up.^85,86^

### Hepatitis B infection

Chronic hepatitis B afflicts over 250 million individuals worldwide and is responsible for close to a million deaths per year.^87,88^ Results from two studies suggest FMT may aid hepatitis B virus e-antigen clearance (2/12 who received FMT *versus* 0/15 who did not in one study, and 3/5 FMT recipients *versus* 0/13 who did not in the other) in chronic carriers who fail to respond to anti-viral therapy. However, small sample sizes and the lack of participant randomisation make it difficult to draw any definitive conclusions as to whether FMT has a role in the setting of chronic hepatitis B infection at present.^89,90^ Results from an RCT completed in March 2018 [ClinicalTrials.gov identifier: NCT02689245] are awaited.

### Obesity and the metabolic syndrome

Two recently published trials of capsulised FMT in obesity did not show benefit over control in a range of outcomes including body mass index and insulin sensitivity despite evidence of microbiota modification in FMT recipients questioning the role of FMT in this setting.^91,92^ This contrasts with historical studies which suggested FMT may be able to increase insulin sensitivity and improve glycaemic control, albeit only in the short term.^93^ There are numerous differences between studies including FMT delivery route and donor selection, which could go some way to explaining observed discrepancies, as well as the fact they were all small studies (the largest had 38 patients in total).

Non-alcoholic fatty liver disease is considered a hepatic manifestation of the metabolic syndrome and is associated with increased gut permeability. Allogeneic FMT from lean donors was superior to autologous FMT in improving gut barrier integrity in the short term (6 weeks), but not in increasing insulin sensitivity or reducing hepatic fat deposition.^94^ This was the first study outside of CDI to show a definitive reduction in intestinal permeability post-FMT and has potentially important ramifications with respect to other conditions.

### Systemic inflammatory response syndrome (SIRS)

In critically unwell patients with acute respiratory distress syndrome, shock or intractable diarrhoea, for which no alternate aetiology can be found, it has been proposed that gut dysbiosis is, at least in part, contributing to, or even driving, the disease process. A reduction in bacteria that produce SCFAs and an increase in pathogenic bacteria such as *Enterococcus* and *Escherichia* has been found in patients admitted to intensive care.^95^ These changes may impair gut integrity and promote the release of toxins into the systemic circulation driving an inflammatory response. As previously discussed, FMT has been demonstrated to reverse changes such as these, and improvement in clinical condition post-FMT has been seen in a small number of cases.^96^ Further studies, including RCTs, are required to appreciate if this can truly be attributed to FMT.

### Anxiety and depression

The gut–brain axis is a bidirectional signalling pathway which may be modulated by the gut microbiota. The current evidence base for the effect of FMT in psychiatric disorders (predominantly anxiety and depression) has been previously summarised.^97^ The overwhelming majority of in-human studies have enrolled patients with concurrent IBS^98–102^ or are limited to case reports,^103–105^ with only one study^98^ involving a control group and blinding, making it difficult to draw firm conclusions at present. Improvements were seen in depression, anxiety, quality-of-life and fatigue scores, although the effect may only be transient.

### Autistic spectrum disorder

The high frequency of concurrent GI symptoms in individuals with autistic spectrum disorder (ASD), and their link to disease severity,^106^ has led to the suggestion that gut dysbiosis may be implicated in disease pathogenesis. Detectable differences in microbiota between children with ASD and controls have been identified,^107,108^ but interestingly not in studies with siblings as controls.^109,110^ FMT is proposed to work by increasing bacterial diversity, improving gut wall integrity and modulating blood metabolites.^111^ An open-label study which recruited 18 children with ASD and 20 age-matched ‘neurotypical’ controls showed improvement in both GI and behavioural symptoms post-FMT delivery. Limitations of the study include the use of antibiotics prior to FMT delivery (which has previously been shown to modulate symptoms^112^), the intensity of the treatment period (10 weeks), short-term follow up (8 weeks), and the lack of randomisation or blinding.

### Parkinson’s disease

Parkinson’s disease (PD) is a progressive neurodegenerative disorder affecting neurons within the central, enteric, and peripheral autonomic nervous systems. Concurrent GI disturbance, usually constipation or slow transit, is common, and may precede motor symptoms.^113^ This has led to the proposition that the disease may even originate in the gut. A key component of disease aetiology is the aggregation of the protein, alpha-synuclein, a major component of Lewy bodies, in these neurons. It has been demonstrated (albeit in animal models) that alpha-synuclein is capable of being transported from the gut to the brain.^114^ This process is thought to be potentiated by gut inflammation.^115^ Numerous changes in the gut have been identified in patients with PD, including increased abundance of pro-inflammatory cytokines and bacteria,^116^ fewer anti-inflammatory-producing bacteria,^115^ and increased intestinal permeability,^117^ supporting this hypothesis. And, as discussed, FMT has the capacity to reverse these changes. Published in-human studies of FMT in PD to date are limited to a single case report, which showed a transient benefit in motor symptoms, but a more prolonged improvement in constipation.^118^ The results of presently recruiting clinical trials will be required to assess in FMT has a future role in PD.

### Epilepsy

Differences in the microbiota of patients with treatment-refractory epilepsy, drug-sensitive epilepsy and healthy controls have been identified, and proven interventions which modulate the host microbiota (ketogenic diet) have been used to manage epilepsy.^119,120^ Studies show a relative increase in abundance of *Firmicute*s compared with *Bacteroidetes* in patients with treatment-refractory disease.^120–122^ *Firmicutes* may alter neurotransmitter levels, which could have an influence on seizure threshold. Higher levels of *Bifidobacteria* and *Lactobacillus* were associated with a reduced seizure frequency.^122^ In humans, studies are limited to a single case report of a patient with concurrent CD. Post-FMT there was a reduction in seizure frequency and an improvement in the activity index of the patient’s IBD.^123^

### Multiple sclerosis

It has been suggested that patients with multiple sclerosis (MS) have a gut microbiota that is less able to induce T_reg_ cells leading to a rise in Th_1_ and Th_17_.^124^ Elevated Th_1_ and Th_17_ cells are in turn hypothesised to induce central nervous system (CNS) inflammation and reduce blood–brain barrier permeability, potentiating further CNS inflammation.^125^ Modulation of the gut microbiota to induce more T_reg_ cells could result in less activation of pathogenic T cells.^126^ Human studies so far have been restricted to case reports/series,^127,128^ and although they show promise, the results of ongoing clinical trials are awaited to understand if FMT will have a role in the management of MS.

### Acute myeloid leukaemia

The combination of intensive chemotherapy and multiple courses of broad-spectrum antimicrobials for febrile neutropaenia result in significant gut dysbiosis in individuals with acute myeloid leukaemia. Autologous FMT harvested pre-treatment and given following induction treatment has been demonstrated to restore microbial diversity and potentially reduce systemic inflammation and the expression of antimicrobial-resistance genes. The study did not include a control group and there was a per-protocol analysis;^129^ nevertheless, results are encouraging, and RCTs are warranted to investigate further.

## Future perspectives

### Delivery route, dosage, and preparation techniques

The future role of FMT is likely to be shaped by how it can be delivered. Capsulised FMT has major advantages over fresh/frozen FMT delivered *via* colonoscopy, not least in terms of patient acceptability, but also practicality and scalability. This comes with the proviso that the active product is not detrimentally affected by the lyophilisation and/or encapsulation process and remains efficacious. There are further advantages if repeated dosing is required to achieve primary efficacy or durable effect, including delivery of therapy as an outpatient (the patient could store their treatment at home negating repeat trips to hospital). Results have emerged to suggest differences in efficacy for different preparations with respect to the indication for which FMT is being provided, for example, FMT appears to be effective *via* all routes/forms for CDI, but not for IBS.

Currently, there is a lack of clarity as to what dose of FMT is necessary to have therapeutic effect, and again, this may be indication dependent.^130^ A minimum of 30 g of faeces has been recommended for the treatment of rCDI.^131^ Further studies are necessary to identify minimum effective dose and redosing frequency for other indications.

Additionally, how FMT should be prepared remains subject to debate. Most colonic bacteria are obligate anaerobes, making the oxygen-rich environment of the outside world a suboptimal climate in which to preserve their viability. Therefore, anaerobic stool preparation has been trialled, with evidence of improved preservation of certain bacterial species using this technique.^132^ Although success rates in rCDI are unchanged by preparatory environment, it may be relevant for other interventions.^133^

### Personalised therapy and standardisation

Recognition that success rates of FMT for indications other than CDI may be donor dependent has resulted in the idea that donor selection could influence FMT outcomes. Many of the stool donations in the described RCTs came from only one or two selected donors. This is beneficial in terms of creating a more universal product, but on the other hand, may mean that results are not widely applicable, and, if greater alpha diversity contributes to the efficacy of FMT, this approach may actually limit treatment effect. At present, there are many ideas about who is the ideal donor. They include a donor who was breastfed when an infant, is a non-smoker, is unrelated to recipient, has had minimal or no previous antibiotic exposure, has no recent travel history to an area with high rates of AROs, and so forth. Presently, there remains little hard evidence to confirm exactly what constitutes a ‘healthy microbiome’, and who is the model donor, and whether having multiple donors is, in fact, superior.

There is currently no licensed, industry-developed FMT-like product that can be administered to patients (outside of a clinical trial). Thus, we are reliant on stool banks set up by healthcare organisations, academia and in some cases, not-for-profit companies, together with the generosity of donors. This model is limited by strict screening criteria and a lack of stool banks to produce material for both routine clinical service and research studies.

Understandably creation of a ‘synthetic’ FMT is a desired goal in terms of reproducibility, safety, regulation and scalability, as well as commercial opportunity. Results of ‘manufactured’ FMT to date have shown promise,^134,135^ and further trials are ongoing [ClinicalTrials.gov identifiers: NCT03244644, NCT03788434 and NCT04208958 to list a few], but there remains a way to go before we are likely to see these entering widespread practice.

### Other potential indications

Hypotheses, *in vitro* work, animal models, and in-human studies yet to be reported have suggested a potential role in a number of indications that we have not discussed in this article. These include, but are not limited to, Alzheimer’s disease, Guillain–Barré syndrome, stroke,^136^ allergy and atopy,^137^ psoriatic (and other inflammatory) arthropathy,^138^ colorectal cancer,^139^ and even COVID-19.^140^

### Clinical trials

Supplementary Table 1 summarises trials in which FMT is being used as the investigational medicinal product (outside of CDI) that have completed, but for which no reported results could be identified. Supplementary Table 2 summarises the ongoing trials using FMT as of 17 August 2020 on ClinicalTrials.gov. The most common indication is IBD (particularly UC), with multiple RCTs with a target recruitment of >100 participants. There are also RCTs with >100 planned participants investigating FMT in eradication of AROs, IBS, cirrhosis, and gut dysbiosis post-Caesarean section and post-stem-cell transplant. Follow up in certain studies is for up to 10 years. These are all important steps in right direction to establishing a solid evidence base for the role of FMT beyond rCDI. Twenty-five trials yet to start recruiting include the additional indications of systemic sclerosis [ClinicalTrials.gov identifier: NCT04300426], alopecia [ClinicalTrials.gov identifier: NCT04238091] and hypertension [ClinicalTrials.gov identifier: NCT04406129].

## Conclusion

Ingestion of faecal material has been used in as medicinal therapy for thousands of years, but it is not until the last decade that high-quality evidence to support the practice of FMT has emerged. FMT has multiple plausible mechanisms of action, including colonisation resistance, anti-inflammatory and immunomodulatory actions and direct antimicrobial properties from transmitted substances such as bacteriocins and organisms such as bacteriophages. However, the relative contribution of each proposed mechanism remains unclear, and probably varies according to the condition being treated.

There is a growing evidence base for the role of FMT for non-CDI indications; however, at present, the overwhelming majority of studies are limited to case reports/series or small pilot studies, and no firm conclusions as to the efficacy of FMT can be drawn. Carefully designed RCTs (many of which are in progress) will be necessary to truly begin to understand what future role, if any, it may play. Further work will also be required with respect to dosing, donor selection, and comparison of delivery routes and FMT preparations. Nevertheless, the early indications are there that FMT could be a promising therapy with established biological plausibility in IBD, hepatic encephalopathy, and a multitude of other conditions. It truly is an exciting and fascinating time to be working on the gut microbiota and faecal transplantation.

## Supplemental Material

sj-pdf-1-tai-10.1177_2049936120981526 – Supplemental material for The role of faecal microbiota transplantation: looking beyond Clostridioides difficile infection Click here for additional data file. Supplemental material, sj-pdf-1-tai-10.1177_2049936120981526 for The role of faecal microbiota transplantation: looking beyond Clostridioides difficile infection by Simon D. Goldenberg and Blair Merrick in Therapeutic Advances in Infectious Disease
