# Supplementary material for: The role of faecal microbiota transplantation: looking beyond Clostridioides difficile infection
Source: Ther Adv Infect Dis. 2021 Jan 25;8:2049936120981526. doi: 10.1177/2049936120981526 (PMC7841662; doi:10.1177/2049936120981526)
Supplement: sj-pdf-1-tai-10.1177_2049936120981526 – Supplemental material for The role of faecal microbiota transplantation: looking beyond Clostridioides difficile infection [file sj-pdf-1-tai-10.1177_2049936120981526.pdf]

Supplementary table 1: Completed, but not yet published, FMT trials (not including those for CDI)

Condition	Identifier	Official title	Participants	Intervention	Primary outcome measure(s)	Follow-up period
Inflammatory bowel disease (ulcerative colitis)	NCT02330653	A Phase I/II, Double Blinded, Placebo Controlled, Single-centre Study of Faecal Microbiota Transplantation (FMT) for the Treatment of Active Paediatric Ulcerative Colitis	60 (actual = 11)<br>5-30 years old	Retention enema followed by capsule FMT vs. placebo	• Safety and tolerability of universal donor FMT compared to placebo: FMT-related adverse events grade 2 or above	1 year
	NCT03378921	Double-blinded Randomized Placebo Controlled Study: Faecal Microbiota Transplantation in the Treatment of Chronic Pouchitis	26<br>18-75 years old	2 doses of allogeneic vs autologous FMT via flexible sigmoidoscopy 4 weeks apart	• Clinical remission	54 weeks
	NCT02428361	Faecal Microbiota Transplant (FMT) for Pouchitis	19<br>18+ years old	250 mL of the faecal suspension in aliquots of 50-60 mL via endoscope	• Clinical improvement of pouchitis	6 months
	NCT04294615	Clinical Response and Safety Following Faecal Microbiota Transplantation by Automatic Methods in Patients with Moderate-Severe Active Ulcerative Colitis	9<br>18-75 years old	Nasojejunal automatically prepared FMT vs. FMT via colonoscopy	• Clinical response	2 weeks
	NCT02049502	The Use of Faecal Microbiota Transplantation in Patients with Ulcerative Colitis-associated Pouchitis	8<br>18-65 years old	FMT via flexible sigmoidoscopy	• Number of Patients Who Experienced Improvement of Pouchitis Symptoms	3 months
	NCT03711006	The Effect of Faecal Microbiota Transplantation in Ulcerative Colitis - A Case Study	7<br>18-50 years old	25 multi-donor FMT capsules daily for 50 days	• Change in patient colitis symptoms as assessed using the Simple Colitis Clinical Activity Index (SCCAI)	24 weeks
	NCT01947101	A Phase I Study of Faecal Microbiota Transplantation (FMT) in Immunomodulator Dependent Paediatric Ulcerative Colitis (UC)	6<br>12-20 years old	FMT endoscopically administered directly into the colon. Consecutive treatments by rectal enema	• Assess safety of FMT treatment by recording the frequency of adverse events.	1 year
	NCT02227342	A Prospective, Single Centre, Open-Label Trial of Faecal Microbiota Transplantation (FMT) in the Management of Active Ulcerative Colitis	3<br>18-65 years old	Serial FMT	• Mayo score reduction	32 weeks
Inflammatory bowel disease (Crohn's disease)	NCT03194529	Safety of FMT in Maintenance of Paediatric Crohn's Disease	9<br>7-21 years old	Equivalent of 50g FMT via nasojejunal route	• Incidence of Treatment-Emergent Adverse Events	24 weeks

	NCT02199561	A Prospective, Single Centre, Open Label Trial of Faecal Microbiota Transplantation (FMT) in the Management of Active Crohn's Disease	3<br>18-65 years old	FMT by colonoscopy at 0, 4 & 8 weeks, and enema at 2 & 6 weeks	• Harvey-Bradshaw index (HBI) score reduction	32 weeks
Inflammatory bowel disease (microscopic colitis)	NCT03275467	Faecal Microbiota Transplantation in Patients with Microscopic Colitis	10<br>18-65 years old	FMT via colonoscopy then enema for 2 further doses	• Proportion of MC patients in remission six weeks after the first FMT	6 months
Inflammatory bowel disease	NCT02487238	A Single-Blind, Randomized, Placebo-Controlled Trial of Human Faecal Microbiota Transplantation for the Therapy of Paediatric Ulcerative Colitis and Inflammatory Bowel Disease Unclassified	35<br>3-17 years old	FMT vs. placebo via enema twice weekly for 6 weeks	• Feasibility (Composite Measure)	30 weeks
Irritable bowel syndrome	NCT02847481	A Pilot Study to Evaluate Faecal Microbiota Transplantation Engraftment in Irritable Bowel Syndrome	80<br>18+ years old	FMT with antibiotic pre-treatment vs. placebo	• Stable engraftment of donor microbiota	10 weeks
Constipation	NCT03308461	Faecal Microbiota Transplantation for Constipation with Difficult Defecation	22<br>14-80 years old	Single dose FMT via endoscope	• Defecation frequency	12 weeks
Diarrhoea	NCT04040712	Faecal Microbiota Transplantation to Treat Diarrhoea Induced by Tyrosine-kinase Inhibitors in Patients with Metastatic Renal Cell Carcinoma: a Randomized Clinical Trial	20<br>18+ years old	FMT vs placebo	• Rate of patients who experience resolution of diarrhoea 4 weeks after the end of treatments	8 weeks
Antibiotic resistant organisms	NCT03063437	Phase II Randomized, Double Blind, Placebo-controlled, Parallel Group Trial of Encapsulated Faecal Microbiota Transplantation for Vancomycin Resistant Enterococcus Decolonization	9<br>18+ years old	30 capsules of FMT vs. placebo	• Percentage of Participants with VRE Decolonization<br>• Percentage of Participants with an Adverse Event (AE); Severe Adverse Event (SAE); and Newly Acquired Transmissible Infectious Diseases Which Are Considered Adverse Events of Special Interest (AESI)	6 months
	NCT03061097	Randomized Controlled Trial of Autologous Microbiome Reconstitution to Prevent Colonization by Antibiotic Resistant Bacteria	4<br>18+ years old	Autologous FMT vs. normal saline via enema	• Safety (short-term) defined as absence of NIH Grade ≥2 adverse events including IND safety reporting criteria	6 months
Metabolic syndrome	NCT03727321	Faecal Microbial Transplantation and Fibre Supplementation in Subjects with Obesity and Metabolic Syndrome	68<br>18-65 years old	50g FMT in capsules vs. placebo	• Insulin Sensitivity Assessment	12 weeks

	NCT02496390	Transplantation of Microbes of Faecal Origin for Prevention and Treatment of Metabolic Syndrome and Non-Alcoholic Fatty Liver Disease	21<br>18+ years old	Allogeneic vs. autologous FMT	• Improvement in Homeostasis model assessment (HOMA) score	6 months
	NCT02730962	Interventional Bioremediation of Microbiota in Metabolic Syndrome	12<br>18-70 years old	Antibiotic pre-treatment or placebo followed by FMT via colonoscopy	• Insulin sensitivity measured by standard euglycemic insulin clamp. Post-FMT insulin sensitivity measurements between subjects that receive antibiotic versus placebo conditioning will be compared	10 weeks
HIV	NCT03008941	A Phase I/II Randomized, Double-Blind, Placebo Controlled Study of Repeated Low-Dose Faecal Microbiota Restoration in HIV-Infected Subjects. The REFRESH Study	30<br>18-80 years old	10 capsules FMT followed by 5 capsules weekly for 7 weeks vs. placebo	• Number of participants with treatment-related adverse events as assessed by CTCAE v4.0	48 weeks
	NCT04165200	Faecal Microbiota Transplantation as a Therapeutic Strategy for Patients Infected With HIV	22<br>18+ years old	60 FMT vs. placebo capsules at 5 time points over 12 weeks	• Levels of CD4 cells	6 months
	NCT03329560	Safety of Faecal Microbiota Transplant using Oral Encapsulated PRIM-DJ2727 in HIV-infected Persons on Antiretroviral Therapy	6<br>18+ years old	Oral capsules containing lyophilized microbiota weekly for 6 weeks	• Number of participants with adverse events related to study drug<br>• Cumulative of adverse events related to study drug<br>• Severity of adverse events related to study drug	1 year
Chronic kidney disease	NCT04361097	Faecal Microbiota Transplantation as a Therapeutic Strategy in the Progression of Chronic Kidney Disease	28<br>18+ years old	60 capsules FMT over 48hrs on days 0, 10 and 30 vs. placebo	• Creatinine Clearance	6 months
Parkinson’s disease		Faecal Microbiota Transplantation as a Potential Treatment for Parkinson's Disease: A Pilot Study	15 (actual= 10)<br>50+ years old	Single dose FMT	• Motor parkinsonian symptoms<br>• Constipation level	6 months
Urinary tract infections	NCT03050515	Faecal Microbiota Transplantation for the Treatment of Recurrent Urinary Tract	12<br>18-100 years old	FMT via retention enema	• Change in frequency of culture proven urinary tract infections following faecal transplant	6 months
Sjögren’s syndrome	NCT03926286	Faecal Microbial Transplantation for Sjögren’s Syndrome	10<br>18+ years	2 doses of FMT enema 1 week apart	• Number of participants with reported adverse events (AEs) and serious adverse events (SAEs)<br>• Number of participants with stable microbiome engraftment	7 months
Hepatitis B infection	NCT02689245	Randomized Controlled Trial Comparing the Efficacy and Safety of FMT in	64<br>18-80 years old	FMT via nasojejunal tube vs. control	• Transplant free survival	3 months

		Hepatitis B Reactivation Leads to Acute on Chronic Liver Failure				
Hepatic encephalopathy	NCT02255617	A Prospective, Single Centre, Open-Label Trial of Faecal Microbiota Transplantation (FMT) in the Management of Hepatic Encephalopathy (HE): a Pilot Study	4<br>18-65 years old	FMT via colonoscopy at week 0 and by enema at weeks 1-4	• Time to hepatic encephalopathy breakthrough	6 months
Mesothelioma	NCT04056026	A Single Dose FMT Infusion from a Healthy Family Donor Via Colonoscopy as an Adjunct to Keytruda for the Benefit of Improving Efficacy of Immunotherapy for Metastatic Mesothelioma	1	FMT via colonoscopy	• Progression free survival	5 years

Supplementary table 2: Ongoing FMT trials (not including those for CDI)

Condition	Identifier	Official title	Participants	Intervention	Primary outcome measure(s)	Follow-up period
Inflammatory bowel disease (ulcerative colitis)	NCT01790061	Efficacy, Durability and Safety of Standardized Faecal Microbiota Transplantation in Patients with Moderate to Severe Ulcerative Colitis	500<br>6-80 years old	FMT via endoscopy	• Efficacy of FMT	10 years
	NCT03483246	Impact of Faecal Microbiota Transplant in Ulcerative Colitis: a Randomized, Sham Controlled Trial	150<br>18-75 years old	FMT vs. placebo via colonoscopy	• Steroid-free clinical and endoscopic remission	24 weeks
	NCT03998488	A Randomized, Placebo-controlled Clinical Trial Examining the Efficacy of Faecal Microbiota Transplantation (FMT) and Subsequent Dietary Fibre in Patients with Moderate Ulcerative Colitis	135<br>18-89 years old	250ml FMT vs. placebo via colonoscopy followed by alternate at 8 weeks +/- fibre	• Clinical Response	8 weeks
	NCT03078803	A Prospective Multicentre Randomized Controlled Trial Comparing Faecal Microbiota Transplantation (FMT) to Placebo in the Treatment of Mild to Moderate Crohn's Disease	126<br>18+ years old	FMT via colonoscopy then oral capsules vs. placebo	• Clinical and Endoscopic Remission	8 weeks
	NCT03804931	Efficacy and Safety of Faecal Microbiota Transplant for Ulcerative Colitis	120<br>18-65 years old	FMT vs. placebo via colonoscopy	• Clinical remission in patient as assessed using Mayo score<br>• Clinical improvement in patient as assessed using Mayo score	12 weeks
	NCT03273465	Faecal Microbiota Transplantation in Newly Diagnosed Patients with Ulcerative Colitis - a Double Blind, Placebo-Controlled Trial	100<br>18-50 years old	FMT vs. placebo via capsules	• UC remission	12 months
	NCT03582969	High Intensity, Multi-Donor, Oral Capsulized Faecal Microbiota Transplantation in Newly Diagnosed Paediatric Patients with Mild to Moderate Ulcerative Colitis - a Double Blind, Placebo-Controlled Trial	100<br>12-18 years old	FMT vs. placebo capsules	• UC remission	1 year
	NCT02606032	Randomized Trial of Antimicrobials Versus Placebo in Addition to Faecal Microbiota Therapy in for the Induction of Remission in Active Ulcerative Colitis	80<br>18+ years old	Antibiotics or placebo followed by FMT twice weekly for 8 weeks	• The primary outcome of the randomized trial is remission of UC defined as a Mayo score < 3 with an endoscopic Mayo score = 0 at the end of the trial Transplant Therapy in Ulcerative Colitis	9 weeks

	NCT04100291	The Effect of Faecal Microbiota Transplantation in the Treatment of Chronic Pouchitis: A Multicentre, Placebo-controlled, Randomized, Double Blinded Trial	50<br>18+ years old	FMT vs. placebo via enema daily for 4 weeks	• Number of patients achieving clinical remission assessed by PDAI	12 months
	NCT03561532	Faecal Microbiota Transplantation in the treatment of ulcerative colitis	48<br>18-75 years old	FMT vs. placebo via colonoscopy	• Maintenance of remission of ulcerative colitis	1 year
	NCT03524352	Prospective Multi-centre Randomized Controlled Double-blind Label Study of the Prophylaxis of Recurrent Pouchitis After Faecal Microbiota Transplant in UC With Ileo-anal Anastomosis	42<br>18+ years old	FMT vs. placebo	• Number of days between the date of transplantation and the date of relapse according to physiological and endoscopic parameter (pouchitis disease activity index)	2 years
	NCT03006809	Optimal Faecal Microbiota Transplant Dosing for Mild to Moderate Ulcerative Colitis	40<br>18-64 years old	4 arms involving pre-treatment with antibiotics vs. none followed by FMT via colonoscopy followed by FMT maintenance via enema or capsules	• The occurrence of a Serious Adverse event (SAE), solicited and unsolicited AE, new gastrointestinal medical condition and diagnoses from FMT treatment or new infection from FMT treatment<br>• Steroid-free Clinical Remission at week 9 + endoscopic remission or response defined as total Mayo score ≤2 with all four sub-scores ≤1 and a ≥1-point reduction in endoscopy sub-score	1 year
	NCT03545386	Randomized Trial of Faecal Microbiota Transplantation Versus Placebo for the Induction of Remission in Patients with Active Pouchitis	34<br>18-99 years old	FMT vs. placebo via enema	• Remission of pouchitis, defined as a pouchitis disease activity index (PDAI) score of <7, with a decreased from baseline PDAI score of 3 points	7 weeks
	NCT03948919	Low Sulphur Faecal Transplant for Ulcerative Colitis	20<br>18-89 years old	FMT vs. placebo via capsule daily for 8 weeks	• Engraftment of low sulphate reducing microbiota	12 weeks
	NCT03716388	Faecal Microbiota Therapy Vs 5-aminosalicylates for Induction of Remission in Newly Diagnosed Mild-moderately Active UC: a Pilot Study	20<br>18-75 years old	FMT vs. placebo +/- mesalamine via colonoscopy at weeks 0, 2, 6, 10, 14	• Clinical remission	14 weeks
Inflammatory bowel disease (Crohn's disease)	NCT01793831	Efficacy and Safety of Standardized Faecal Microbiota Transplantation for Moderate to Severe Crohn's Disease	200<br>6-80 years old	Not specified	• Clinical remission	10 years

	NCT03378167	PediCRaFT: Paediatric Crohn's Disease Faecal Microbiota Transplant Pilot Study	45<br>3-17 years old	FMT vs. placebo via colonoscopy followed by FMT vs. placebo capsules for 6 weeks	• Monthly Recruitment Rate<br>• Dropout Rate Post Enrolment<br>• Rate of Patient Protocol Adherence<br>• Rate of Adverse Events	30 weeks
	NCT02417974	Prevention of Recurrence of Crohn's Disease by Faecal Microbiota Therapy (FMT)	44<br>18+ years old	FMT vs. placebo via colonoscopy	• Post-operative endoscopic recurrence	6 months
	NCT02897661	Washed Microbiota Transplant Combining Exclusive Enteral Nutrition Contribute to Nutritional Improvement of Patients with Crohn's Disease	30<br>18-65 years old	Early vs. delayed washed FMT	• changes in haemoglobin<br>• changes in albumin and prealbumin in g/L<br>• changes in lymphocyte count in 10^9/L	15 days
	NCT03267238	Faecal Microbial Transplantation in Patients with Crohn's Disease	9<br>7+ years old	Not specified	• Number of participants with treatment-related adverse events as assessed by CTCAE v4.0.	6 years
Inflammatory bowel disease	NCT04436874	Faecal Microbiota Transplantation in the Treatment of Inflammatory Bowel Disease: the Role of Selection of the Dai Ethnic Group as Donor Source in Terms of Treatment Efficacy and Pathogenic Mechanisms	240<br>18-75 years old	FMT via endoscopy	• Steroid-free clinical remission and endoscopic remission	3 years
	NCT03399188	Faecal Microbiota Transplantation and Analysis of Faecal Microbiome in IBD Patients	100<br>7-80 years old	FMT via colonoscopy	• Clinical remission	6 months
	NCT04011943	Study of the Effects of Faecal Microbiota Transplantation for Correction of the Physiological State of the Human Body in Norm and in Pathology	50<br>18-75 years old	Allogeneic vs. autologous FMT vs. placebo	• Ulcerative Colitis remission<br>• Crohn Disease remission	1 month
	NCT02734589	Faecal Transplantation Using a Novel Conditioning Method for Donor and Recipient in Mild to Moderate Treatment Refractory Colitis in Inflammatory Bowel Disease	34<br>18-70 years old	FMT via endoscopy or colonoscopy from donor who has vs. has not undertaken special diet vs. control	• Clinical remission	12 weeks
Antibiotic resistant organisms	NCT04188743	DEKODON: Decolonization of Gram-negative Multi-resistant Organisms (MDRO) With Donor Microbiota (FMT)	150<br>18+ years old	Allogeneic vs. autologous FMT via endoscopy vs. control	• Number of participants with decolonization success/failure	1 year
	NCT04181112	Faecal Microbiota Transplantation (FMT) for Intestinal Decolonization of Multidrug-resistant Opportunistic Pathogens	90<br>18+ years old	FMT via enema +/- antibiotic pre-treatment	• The elimination of the target multi-drug resistant organism (MDRO), using culture and	3 years

					molecular test-based screening of recipient stool, at both the 14 and 30 days post-FMT.	
	NCT03367910	Faecal Microbiota Transplantation Using RBX2660 for the Prevention of Recurrent Urinary Tract Infections Due to Multidrug Resistant Organisms	60<br>18+ years old	FMT via enema	• FMT safety: adverse events during and after FMT	6 months
	NCT03029078	Faecal Microbiota, a Hope to Eradicate Colonization of Patient Harbouring eXtreme Drug Resistant Bacteria?	50<br>18+ years old	FMT via nasoduodenal tube	• Negativation of digestive tract colonization	6 months
	NCT03802461	Effectiveness of Faecal Flora Alteration for Eradication of Carbapenemase-producing Enterobacteriaceae Colonization Trial (EFFECT-CPE): a Multisite, Open-label, Randomized Controlled Feasibility Pilot Trial	40<br>18+ years old	3 doses of 50g FMT vs. control	• Incidence of intestinal colonization of patients with CPE 3 months after intervention.<br>• Randomization rate in study<br>• Proportion of patients retained in study for up to 6 months	12 months
	NCT03479710	Faecal Microbiota Transplantation for Eradication of Intestinal Colonization of Carbapenem-resistant Enterobacteriaceae and Vancomycin-resistant Enterococcus: a Pilot Study	40<br>18+ years old	FMT via endoscopy vs. control	• Intestinal colonization of CRE/VRE	12 months
	NCT02922816	A Pilot Study Using Faecal Microbiota Transplant in Renal transplant Recipients to Eliminate Multidrug-Resistant Organism Colonization After Infection and Compare Gastrointestinal Carriage in a Randomized, Controlled, Crossover Design	20<br>18+ years old	FMT via rectal enema for 2 doses	• The safety and feasibility of using FMT in adult participants with Target MDRO colonization after infection will be measured by comparing the number of adverse events (assessed by CTCAE v4.0) after Day 1 of each cycle as compared to baseline.	30 weeks
	NCT02312986	Use of Faecal Microbiota Transplantation (FMT) to Reverse Multi-Drug Resistant Organism Carriage	20<br>18+ years old	FMT via enema	• Safety of FMT in patients with recurrent MDRO infections	1 year
	NCT02543866	Faecal Microbiota Transplantation as a Strategy to Eradicate Intestinal Carriage of Resistant Organisms	20<br>7-21 years old	FMT via nasogastric tube	• Safety and Tolerability of FMT as measured by Incidence, severity, and relatedness of solicited, unsolicited, and serious adverse events	5 years
Metabolic syndrome	NCT03127696	A Randomised Placebo-controlled Study of Faecal Microbiota Transplant (FMT) to Impact Body Weight and Glycaemic Control in Obese Subjects with Type 2 Diabetes Mellitus	61<br>18-70 years old	FMT vs. placebo +/- lifestyle modifications	• Proportion of subjects with at least 20% lean-associated microbiota in recipients after FMT compared with subjects receiving lifestyle intervention alone up to week 24	24 weeks
	NCT03273855	Randomized Controlled Trial of Faecal Microbiota Transplantation in Severe Obesity	60<br>18-69 years old	Allogeneic vs. autologous FMT via enema	• Change in individual weight loss (kg)	12 months
	NCT02970877	Faecal Microbiota Transplant from Healthy Lean Donors to Morbidly Obese Individuals:	48<br>18+ years old	Allogeneic vs autologous FMT	• Change in Insulin Resistance compared to baseline	3 months

		Effect on Insulin Resistance and Other Obesity-related Parameters. A Randomized Controlled Trial.				
	NCT03391817	Faecal Microbiota Transplantation in the treatment of morbid obesity	40<br>18-65 years old	Allogeneic vs. autologous FMT via endoscopy	• Reduction of weight	18 months
	NCT03789461	An Open-label Pilot Study of Faecal Microbiota Transplant (FMT) to Induce Weight Loss in Obese Subjects	20<br>18-75 years old	Not specified	• Proportion of at least 10% reduction in weight	2 years
	NCT04465032	The Effect of Consecutive Faecal Microbiota Transplantation on Non-Alcoholic Fatty Liver Disease (NAFLD) - a Randomized-controlled Trial	20<br>18-70 years old	Allogeneic vs. autologous FMT via endoscopy at 0, 3, 6 weeks	• the effect on consecutive FMT on liver fat accumulation	14 weeks
Type 1 diabetes	NCT04124211	Faecal Microbiome Transplant (FMT) for Type 1 Diabetes	10<br>18-65 years old	FMT via endoscopy	• Changes in mean amplitude of glycaemic excursion (MAGE)<br>• Changes in standard deviation of blood glucose (SDBG)<br>• Changes in haemoglobin A1c (HbA1c)<br>• Safety of FMT	24 weeks
Graft versus host disease	NCT03148743	Faecal Microbiota Transplantation in Gut aGVHD Treated	50<br>10-60 years old	Not specified	• Times of stool	1 week
	NCT03359980	Treatment of Steroid Refractory Gastro-intestinal Acute Graft-versus-Host disEase afteR AllogeneiC Hematopoietic Stem celL Transplantation with fEcal Microbiota tranSfer	32<br>18+ years old	MaaT013 via enema	• Efficacy of FMT in the treatment of Steroid Refractory -Gastro-intestinal Acute GVHD (SR-GI-aGVHD) at D28 post inclusion	6 months
	NCT03812705	Faecal Microbiota Transplantation for the Treatment of Steroid Resistant/Dependent Acute Gastrointestinal Graft Versus Host Disease	30<br>14-60 years old	FMT via endoscopy or colonoscopy up to 5 times	• Response rate	12 weeks
	NCT04285424	Faecal Microbiota Transplantation for Treatment of Steroid Resistant Acute Graft Versus Host Disease of the Gut	30<br>18-75 years old	FMT via endoscopy or colonoscopy	• Gut acute Graft-versus-Host Disease (aGvHD) response	7 days
	NCT04269850	Pilot Study of Faecal Microbiota Transplantation in Combination with Ruxolitinib and Steroids for Severe Acute Intestinal Graft-versus-host-disease After Allogeneic Hematopoietic Stem Cell Transplantation	20<br>All ages	FMT via capsule (2 capsules/kg body weight)	• Overall survival	1 year

	NCT03819803	Faecal Microbiota Transplantation in Patients with Acute Gastrointestinal Graft-versus-host-disease After Allogeneic Stem Cell Transplantation	15<br>18-70 years old	FMT via colonoscopy	• GI-aGvHD remission	1 year
HSCT	NCT03678493	A Randomized Placebo-Controlled Clinical Trial of Faecal Microbiota Transplantation in Patients with Acute Myeloid Leukaemia and Allogeneic Hematopoietic Cell Transplantation Recipients	120<br>18+ years old	FMT vs. placebo via capsule	• Efficacy of FMT in AML patients and allo-HCT recipients - Incidence of infections	6 months
	NCT03720392	A Phase 2 Study of Faecal Microbiota Transplantation (FMT) in Recipients After Allogeneic Hematopoietic Cell Transplantation (HCT)	48<br>18-80 years old	FMT vs. placebo via capsule	• The proportion of patients who achieve gut microbiome diversity at one month following the final post-HCT FMT	12 months
Cirrhosis	NCT03796598	Faecal Microbiota Transplant in Veterans with Cirrhosis	100<br>21-75 years old	FMT vs. placebo via enema and FMT vs. placebo via capsule	• Serious adverse events related to FMT	6 months
	NCT02862249	A Prospective, Randomised Placebo Controlled Feasibility Trial of Faecal Microbiota Transplantation in Cirrhosis	32<br>18-75 years old	50g FMT vs. placebo via endoscopy	• Assessment of the feasibility of FMT	18 months
	NCT03420482	Faecal Microbiota Transplant as Treatment of Hepatic Encephalopathy	30<br>18-75 years old	15 capsules of FMT vs. placebo on days 1, 2, 7, 14, 21	• Psychometric Hepatic Encephalopathy Score (PHES)	4 weeks
	NCT03439982	A Prospective Single Centre Open-Label Trial of RBX2660 (Microbiota Suspension) in the Management of Hepatic Encephalopathy	30<br>18+ years old	FMT via colonoscopy (week 0) then weekly by enema (1-4)	• Time to HE breakthrough	9 weeks
Alcoholic hepatitis	NCT03091010	A Comparison of Faecal Microbiota Transplantation and Steroid Therapy in Patients with Severe Alcoholic Hepatitis A Randomized Controlled Trial.	112<br>21-70 years old	FMT via nasojejunal tube vs. standard of care	• Proportion of participants with Overall Survival at 3 months	6 months
	NCT03827772	Faecal Microbiota Transplantation in Severe Alcoholic Hepatitis- Assessment of Impact on Prognosis and Short-term Outcome	40<br>18-60 years old	30g FMT via nasojejunal tube vs. control	• Survival	3 months
Hepatitis B infection	NCT04431375	Efficacy of Addition of Faecal Microbiota Transplant (FMT) and Plasma Exchange to Tenofovir in Comparison to Monotherapy with Tenofovir in ACLF-HBV	70<br>18-75 years old	FMT (and plasma exchange) vs. control	• Overall survival in both groups	3 months

	NCT03429439	A Randomised Study on Intestinal Microbiota Transplantation for Chronic Hepatitis B Combined with Antiviral Therapy	60<br>18-65 years old	FMT via endoscopy every 2 weeks for 6 doses	• Change of serum hepatitis B virus e antigen (HBeAg) level	6 months
Irritable bowel syndrome	NCT04236843	Faecal Microbiota Transplantation (FMT) in Patients with Irritable Bowel Syndrome (IBS): Optimizing the Treatment and Its Mechanism(s) of Action	150<br>18-75 years old	90g FMT delivered once or twice into small intestine or once into large intestine	• Change in IBS-SSS total score	3 months
	NCT03613545	Efficacy and Safety of Faecal Microbiota Transplantation for Irritable Bowel Syndrome	120<br>18-75 years old	FMT vs. placebo via colonoscopy	• Change in Irritable Bowel Syndrome assessed by Symptom Severity Score (IBS-SSS)	6 months
	NCT03074227	The FAIS-Trial: Faecal Transplantation in Adolescents with Refractory Irritable Bowel Syndrome	30<br>16-21 years old	Allogeneic vs. autologous FMT via endoscopy	• The proportion of patients with > 50% reduction of their abdominal pain intensity and pain frequency at t=12 weeks after the first faecal transplantation	12 months
	NCT02651740	Efficacy and Safety Evaluation of Rifaximin Combined Faecal Microbiota Transplantation in the Treatment of Irritable Bowel Syndrome with Predominant Diarrhoea	10<br>18-65 years old	FMT via enteral tube at least once	• Number of patients with relief of IBS condition	6 months
Autistic spectrum disorder	NCT03408886	Microbiota Transfer Therapy for Adults with Autism Spectrum Disorder (ASD) Who Have Gastrointestinal Disorders	84<br>18-60 years old	FMT vs. placebo via capsules	• Change in Childhood Autism Rating Scale (CARS) from baseline to 10 weeks	18 months
	NCT04182633	Microbiota Transfer Therapy for Children with Autism Spectrum Disorder (ASD) Who Have Gastrointestinal Disorders	70<br>5-17 years old	FMT vs. placebo via capsule for 12 weeks	• Childhood Autism Rating Scale (CARS)<br>• Gastrointestinal Symptom Severity Scale (GSRS)	18 months
	NCT04142255	A Clinical Study to Explore the Efficacy of Faecal Microbiota Transplantation (FMT) in the Treatment of Childhood ASD Patients with Gastrointestinal Symptoms	20<br>3-17 years old	Age based dosing of FMT daily for 3 days then weekly for 3 weeks	• Gastrointestinal Symptom Rating Scale (GSRS)<br>• Childhood Autism Rating Scale (CARS)	24 weeks
	NCT03426826	Dynamics of Gut Microbiomes in Autism Spectrum Disorder (ASD) Symptoms	10<br>5-17 years	FMT vs. placebo via endoscopy	• Primary Outcome Measures, safety and tolerability	24 weeks
Chronic fatigue syndrome	NCT03691987	Faecal Microbiota Transplantation in Chronic Fatigue Syndrome - an RCT	80<br>18-65 years old	50-80g Allogeneic vs. autologous FMT via enema	• Change in individual global Fatigue Severity Scale score.	12 months

	NCT04158427	Intestinal Microbiota and Chronic Fatigue Syndrome. Effect of Faecal Transplant on Health-Related Quality of Life of the Patients with Chronic Fatigue Syndrome	40<br>18-65 years old	Allogeneic vs. autologous FMT via colonoscopy	• Health related quality of life assessed by EQ-5D-5L questionnaire<br>• Health related quality of life assessed by 15D questionnaire<br>• Health related quality of life assessed by Modified Fatigue Impact Scale<br>• Ability to work or study<br>• Visual Analog Fatigue Scale	1 month
Pitt Hopkins syndrome	NCT04132427	Microbiota Transfer Therapy for Children with Both Pitt Hopkins Syndrome and Gastrointestinal Disorders	10<br>7-17 years old	FMT vs. placebo via capsules	• Daily stool record<br>• Safety measures	14 weeks
Bipolar disorder	NCT03279224	A Randomized Controlled Trial of the Safety and Efficacy of Faecal Microbiota Transplantation in a Population with Bipolar Disorder	60<br>18-65 years old	50g allogeneic vs. autologous FMT via colonoscopy	• Change in the MADRS total score from baseline (pre-intervention) to the final visit (week 24)	24 weeks
Anorexia nervosa	NCT03928808	Safety and Efficacy of Faecal Microbiota Transplantation (FMT) in the Treatment of Severe and Enduring Anorexia Nervosa (SE-AN)	10<br>18-45 years	FMT via nasogastric tube weekly for 4 weeks	• Proportion of Participants with Adverse Events (AEs)<br>• Proportion of Participants with a Severe Adverse Event (SAE)<br>• Number of Participants Recruited<br>• Number of Participants Able to Complete 4 FMT Administrations<br>• Number of Participants Reporting Acceptable Levels of GI distress Post FMT	3 years
Multiple sclerosis	NCT03594487	Faecal Microbiota Transplantation (FMT) of FMP30 in Relapsing-Remitting Multiple Sclerosis: A Phase 1b Clinical Trial to Evaluate Feasibility, Safety, Tolerability and Effects on Immune Function	30<br>18-60 years	Up to 37.5g FMT via colonoscopy	• Subjects who complete the study protocol<br>• Change in faecal microbiota<br>• Incidence of Treatment-Emergent Adverse Events [Safety and Tolerability]	1 year
	NCT04203017	Allogeneic Faecal Microbiota Transplantation as a Consolidation Treatment After Autologous Hematopoietic Stem Cell Transplantation in Patients with Multiple Sclerosis	20<br>18-55 years old	FMT via capsule	• To evaluate effectiveness of auto-HSCT in combination with FMT in patients with refractory multiple sclerosis	1 year
	NCT03975413	Single-Arm, Non-Randomized, Time Series, Single-Subject Study: Faecal Microbiota Transplantation (FMT) in Multiple Sclerosis	1	Not specified	• Faecal microbial community structure and functional changes over six timeframes for phylum, genus and species taxonomic level bacteria, virus, fungi, and archaea<br>• Walking and balance changes over four timeframes for stride time (seconds)	1 year

					• Walking and balance changes over four timeframes for stride distance<br>• Walking and balance changes over four timeframes for cadence<br>• Walking and balance changes over four timeframes for step width<br>• Walking and balance changes over four timeframes for average pelvis forward velocity<br>• Walking and balance changes over four timeframes for pelvis smoothness	
Parkinson's disease	NCT03808389	A Double-blind, Placebo-controlled, Randomized Clinical Trial Investigating Faecal Microbiota Transplantation for Parkinson's Disease and Its Effect on Symptoms and Disease Progression	40<br>5-65 years old	Allogeneic vs. autologous FMT via nasojejunal tube	• Changes in clinical symptoms as scored on the MDS-UPDRS (Movement Disorder Society - Unified Parkinson's Disease Rating Scale)	1 year
Amyotrophic lateral sclerosis	NCT03766321	Interplay Between Gut Microbiota and Adaptive Immunity in Amyotrophic Lateral Sclerosis: a Clinical Trial	42<br>18-70 years old	FMT via nasojejunal tube vs. placebo at 0 and 6 months	• Change in T_{regs} number	1 year
Epilepsy	NCT02889627	Efficacy and Safety of Faecal Microbiota Transplantation for Epilepsy	30<br>3-70 years old	FMT via endoscopy or colonoscopy	• Frequency of the seizures	3 months
Melanoma	NCT03353402	Altering the Gut Microbiota of Melanoma Patients Who Failed Immunotherapy Using Faecal Microbiota Transplantation (FMT) From Responding Patients	40<br>18+ years	FMT via colonoscopy then capsules	• Incidence of FMT-related Adverse Events<br>• Proper implant engraftment	4 years
	NCT03341143	Phase II Feasibility Study of Faecal Microbiota Transplant (FMT) in Advanced Melanoma Patients Not Responding to PD-1 Blockade	20<br>18+ years old	FMT via colonoscopy	• Objective Response Rate (ORR)	4 years
Prostate cancer	NCT04116775	A Phase II Single Arm Study of Faecal Microbiota Transplant (FMT) in Men with Metastatic Castration Resistant Prostate Cancer Whose Cancer Has Not Responded to Enzalutamide + Pembrolizumab	32<br>18+ years old	FMT every 3 weeks for 5 cycles	• Anticancer effect of faecal microbiota transplant from responders to pembrolizumab to non-responders	2 years
Renal cancer	NCT04163289	Preventing Immune-Related Adverse Events in Renal Cell Carcinoma Patients Treated with Combination Immunotherapy Using Faecal Microbiota Transplantation	20<br>18+ years old	FMT via capsule at 3 time points	• Occurrence of immune-related colitis associated with ipilimumab/nivolumab treatment	9 years

Gastrointestinal cancers	NCT04130763	Investigator-initiated Trial of Faecal Microbiota Transplant (FMT) Capsule for Improving the Efficacy of Anti-PD-1 in Patients With PD-1 Resistant Digestive System Cancers	10<br>18-70 years old	FMT via capsule for 3 days then weekly for 6 weeks	• Objective Response Rate (ORR)<br>• Rate of abnormal vital signs and laboratory test results<br>• The number of adverse events	14 weeks
Gut dysbiosis	NCT04173208	Main Trial of the Caesarean Section and Intestinal Flora of the Newborn Study	100<br>18+ years old	FMT vs. placebo via oral route	• Longitudinal change of intestinal microbiota assessed with 16S rRNA and shotgun sequencing	2 years
Allergy	NCT02960074	A Phase I Open Label Trial to Evaluate the Safety and Efficacy of Oral Encapsulated Faecal Microbiota Transplantation in Peanut Allergic Patients	15<br>18-40 years old	FMT via capsule over 2 days (1/3 to receive antibiotics prior)	• Presence of FMT-related adverse events grade 2 or above	1 year
Malnutrition	NCT03087097	Transfer of Healthy Gut Flora for Restoration of Intestinal Microbiota Via Enema (THRIVE) for Patients in the Rehabilitative Phase of Malnutrition: A Pilot Study Evaluating Microbial Engraftment, Safety and Nutritional Outcome	20<br>1-5 years old	FMT via enema (weight-based dosing)	• Serious Adverse Events	8 weeks
Psoriatic arthritis	NCT03058900	Efficacy and Safety of Faecal Microbiota Transplantation (FMT) in Patients with Peripheral Psoriatic Arthritis: a 6-month, Double-Blind, Randomized, Placebo-Controlled Trial	31<br>18-75 years old	50g FMT vs. placebo via endoscopy	• Treatment failure	6 months
Rheumatoid arthritis	NCT03944096	Efficacy and Safety of Faecal Microbiota Transplantation in Patients with Rheumatoid Arthritis Refractory to Methotrexate: a 24-week, Double-Blind, Randomised Trial	30<br>18-65 years old	50g allogeneic vs. autologous FMT at 0 and 4 weeks via endoscopy	• The American College of Rheumatology 20 (ACR20) response at 16 weeks	24 weeks
Ankylosing spondylitis	NCT03726645	The Role of Gut Microbiota in the Pathogenesis of Ankylosing Spondylitis (AS), and the Effect of Faecal Microbiota Transplantation on Gut Microbiota, Gut Wall Inflammation and Clinical Activity of AS	20<br>18-75 years old	Allogeneic vs. autologous FMT via colonoscopy	• The effect of FMT (Faecal Microbiota Transplantation) on the clinical activity of ankylosing spondylitis (AS) as assessed by change in BASDAI	1 year
IgA nephropathy	NCT03633864	Faecal Microbiota Transplantation for Refractory IgA Nephropathy: a Prospective, Single-centre, Cohort Study	30<br>18-65 years old	FMT via endoscopy or enema	• Change of Urinary protein	8 weeks
Multiple	NCT04014413	Safety and Efficacy of Faecal Microbiota Transplantation: A Pilot Study	450<br>All ages	Not specified	• The efficacy of FMT in treating dysbiosis-associated disorder will be assessed by number of patients who have improvement in clinical	1 year

					symptoms (depends on each disease as stated in outcome)	
	NCT03834051	Prospective, Open-label Trial to Evaluate Efficacy of Faecal Microbiota Transplantation for Treatment of Chronic Gastrointestinal Dysbiosis or Clearance of Antimicrobial Resistant Organism	50<br>18+ years old	FMT via enema	• Symptom improvement in formal diagnosis of dysbiosis or clearance of antimicrobial resistant organism	2 years
	NCT03268213	Faecal Microbial Transplantation in Patients with Medication Refractory Clostridium Difficile and/or Ulcerative Colitis or Indeterminate Colitis	12<br>7+ years old	FMT via colonoscopy	• Number of participants with treatment-related adverse events as assessed by CTCAE v4.0	10 years
